# Parkinsonism in a Patient with Human T-lymphotropic Virus 1 Myelopathy

**DOI:** 10.7759/cureus.2940

**Published:** 2018-07-07

**Authors:** Hasnain Afzal, Satish Kadakia, Steven Lev

**Affiliations:** 1 Resident Physician Psychiatry, Nassau University Medical Center, East Meadow, USA; 2 Chairman Department of Neurology, Nassau University Medical Center, East Meadow, USA; 3 Director of Neuroradiology, Nassau University Medical Center, East Meadow, USA

**Keywords:** ham/tsp, parkinsonism, multiple sclerosis, polymyositis, human t-cell lymphotropic virus, htlv-1

## Abstract

Human T-lymphotropic virus 1 (HTLV-1) infection is commonly associated with neurological conditions like chronic progressive myelopathy and tropical spastic paraparesis (TSP) but rarely also reported with polymyositis, multiple sclerosis, and parkinsonism. It is important to recognize that HTLV-1 infection increases the risk of these neurological conditions. We present a case of 71-year-old female with HTLV-1 associated chronic progressive myelopathy with parkinsonism which signifies that it is under-recognized and not frequently reported due to lack of expert neurological assessment in these chronically debilitated patients.

## Introduction

The human T-lymphotropic virus type 1 (HTLV-1) was discovered in 1980 and is one of the first retroviruses to be discovered. It affects about 10-20 million people around the world. It is transmitted by sexual contact, vertical transmission before birth either transplacentally or during C-section, after birth through breastfeeding [1], contaminated blood needles and blood transfusion [2]. It is associated with a group of hematologic and neurological manifestations. The most common neurological manifestation is HTLV-1-associated myelopathy/tropical spastic paraparesis (HAM/TSP) but the people affected by the virus can also develop various isolated and diverse clinical syndromes including parkinsonism [3]. Parkinson's disease (PD) is the most common neurodegenerative cause of parkinsonism presenting with tremors, slow movement, and stiffness [4]. There are several neurotropic viruses leading to parkinsonism [5]. Parkinsonism is reported in 5%-50% of all acquired immune deficiency syndrome (AIDS) HTLV-III patients [6], but only one isolated case of parkinsonism is reported with HTLV-1 infection [7]. We report a case of 71-year-old female with a long history of HTLV-1 infection with myelopathy and parkinsonism.

## Case presentation

**Figure 1:**
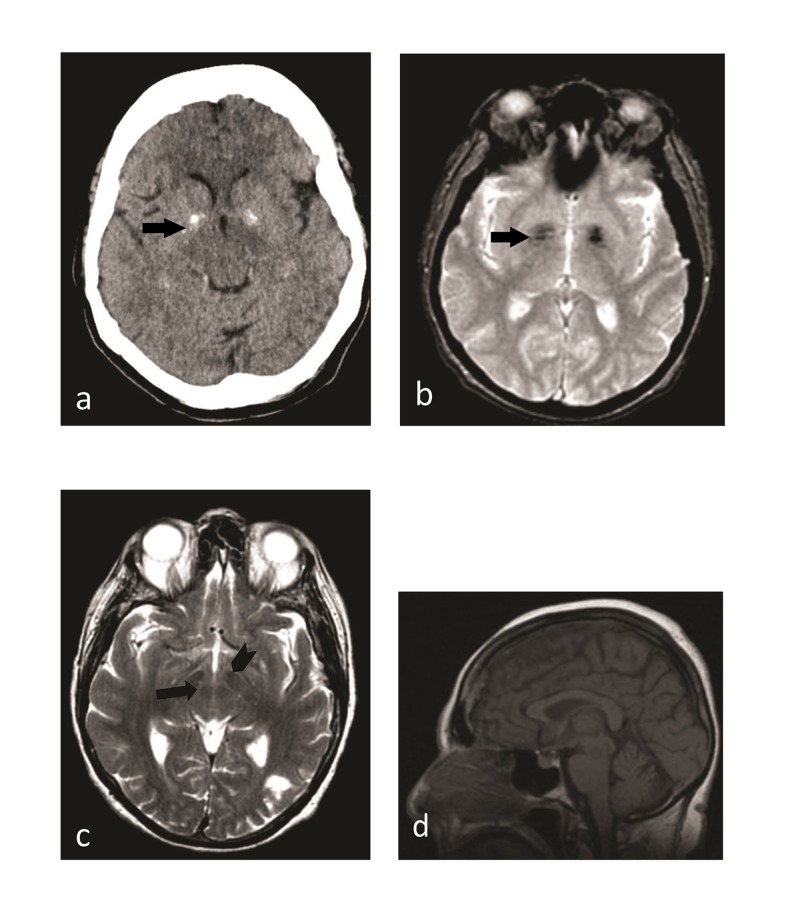
Computed tomography (CT) scan and magnetic resonance imaging (MRI) findings Axial CT image demonstrates (a) mild atrophy and bilateral basal ganglia calcifications (black arrow), which appear hypointense on a corresponding T2W MR image (black arrow); (b) axial T2W image at the level of the midbrain; (c) iron accumulation related hypointensities of the red nucleus (black arrow) and the substantia nigra (black arrowhead). Midline sagittal T1W image shows (d) subtle cortical atrophy, with more pronounced atrophy of the superior vermis.

A 71-year-old female with the history of HTLV-1 infection for 20 years, congestive heart failure, coronary artery disease, hypertension, diabetes type-2, peripheral vascular disease, chronic neck and back pain, nonambulatory for a year admitted to the medical service as dehydration and acute kidney injury. Neurology was consulted for worsening weakness and pain in the legs with paresthesia as well as evaluation for HTLV-1 myelopathy with pain, stiffness and gait problems. Neurological examination showed flat, mask-like face with a positive glabellar reflex. She was noted to have decreased power bilaterally in upper and lower extremities with brisk reflexes and hypertonia. Motor examination of upper extremities showed a strength of 4 x 5 with brisk reflexes and also noted to have resting as well as intention tremor. Lower extremity muscle power was 2 x 5 with brisk reflexes and bilateral clonus and bilaterally upgoing toes. Sensory examination was normal with generalized diffuse rigidity. Her workup included computed tomography (CT) scan of the brain showing bilateral basal ganglia calcifications and mild cortical atrophy, magnetic resonance imaging (MRI) brain T2W image demonstrating low signal intensity from iron accumulation in the red nucleus, and substantia nigra and atrophy of the cerebral cortex and superior vermis of the cerebellum (Figure 1). MRI C-spine, MRI of the thoracolumbar spine and magnetic resonance angiogram (MRA) were unremarkable. Serum HTLV-1 antibody was positive by enzyme immunoassay (EIA) and glutamic acid decarboxylase (GAD65) antibody was also positive at 8 IU/ml by enzyme-linked immunosorbent assay (ELISA). The clinical evaluation was suggestive of HTLV-1 related myelopathy with parkinsonism and patient was started on a trial of baclofen and Sinemet® (carbidopa-levodopa).

## Discussion

Parkinsonism has been commonly reported with HIV (HTLV-III virus) but an extensive literature search using Google Scholar and PubMed highlighted only one case report of HTLV-1 virus infection with myelopathy and parkinsonism [7]. PD is a slowly progressive neurodegenerative disease with symptoms including bradykinesia accompanied by tremor or rigidity. The term parkinsonism is, however, a broader term which refers to the neurological disorders that can have movement problems seen in PD. The diagnostic criteria for PD according to Movement Disorder Society is an expert clinician [8]. There are no blood tests to diagnose PD so, with advances in medicinal technology, it is important to develop predictive and valid biomarkers for diagnosis of PD. Recent researchers have shown the importance of early diagnosis of PD in the prodromal stage, not only to target the pathogenic changes but also slow down disease progression [9]. Although MRI has its limitations in diagnosing PD, it is useful to differentiate PD from atypical parkinsonian disorders. MRI findings such as regional cerebral atrophy and increased iron deposition in the substantia nigra (SN) and red nucleus (RN) have been seen in patients with PD [9-10]. There are several neurological diseases that can present like PD making it difficult to diagnose so, the new guidelines by American Family physicians encourage physicians who rarely diagnose PD to be vigilant in identifying and referring these patients to an expert Neurologist [11].

HTLV-1 infection can be diagnosed with serology, by detection of antibodies in the serum or in the cerebrospinal fluid (CSF) and by polymerase chain reaction (PCR) detection of proviral DNA [12]. In our case, the patient was diagnosed with the HTLV-1 infection two decades ago but we reconfirmed the infection by identifying antibodies in the serum by EIA. Patient's GAD65 antibody was positive at a low titer. GAD65 antibodies are seen in a variety of autoimmune neurologic disorders including encephalitis, myelopathies, myasthenia gravis, Lambert-Eaton syndrome, stiff-man (Moersch-Woltman) syndrome, and autoimmune endocrine diseases like type-1 diabetes, pernicious anemia, premature ovarian failure, Addison disease and thyroid diseases like thyrotoxicosis, Grave disease, Hashimoto thyroiditis and hypothyroidism [13].

The HTLV-1 virus commonly affects the white matter in the thoracic and lumbar segments of the spinal cord but it also affects the brain white matter [14]. The most common myelopathy associated with HTLV-1 is chronic progressive myelopathy and TSP. HAM/TSP is diagnosed by WHO criteria recognizing the symptoms like chronic onset back pain, paraplegia, and urinary problems as well as identifying HTLV-1 antibodies in the serum and CSF [15]. In our case, the patient didn’t have signs of encephalitis, so CSF studies were not done but the patient was given a diagnosis of chronic myelopathy in the setting of HTLV-1 infection. The neuropathogenesis of HTLV-1 infection is not completely understood but it depends upon both the direct effect of the HTLV-1 virus and the immunological response by the immune system [16]. Post-mortem studies have shown that HTLV-1 virus causes inflammation and damage in both brain and spinal [17]. The autopsy of the case of HAM presenting with amyotrophic lateral sclerosis showed degenerative changes in the dorsal columns of the spinal cord, basal ganglia, thalamus and brainstem tegmentum [18]. Morgan et al. (2007) studied 10 MRI of patients with HAM/TSP and 20 MRI of HTV-1 carriers without neurological symptoms and found that 80% of HAM/TSP and 85% of HTLV-I have cerebral white matter changes. These MRI changes do not distinguish HTLV-I carriers from HAM/TSP suggesting that HTLV-1 causes early central nervous system (CNS) inflammation with the possibility of progression [19]. Recent evidence suggests that HTLV-1 can cause encephalopathy and possibly acute encephalitis requiring treatment with IV steroids [20]. In summary, HTLV-1 infection is associated with diverse neurological syndromes affecting both the brain and spinal cord which requires a high index of suspicion on part of the physician due to the rare occurrence. Although limited by the absence of the histopathological evaluation, we suggest with our case that symptoms of parkinsonism in the setting of HTLV-1 infection with associated myelopathy could be likely due to its pathological phenomenon in the brain.

## Conclusions

HTLV-1 commonly affects spinal cord but it can also affect the brain. Clinicians providing care should be vigilant in diagnosing these syndromes associated with HTLV-1 infection. Our patient presented with stiffness, mask-like face, bilateral hand tremors at rest and gait problems, and after a thorough neurological examination was diagnosed with parkinsonism and appropriate treatment was started. We are reporting the case so that these syndromes can be identified and treated appropriately. Future research should be targeted on HTLV-1 pathological changes in the brain so that its pathology can be better understood, improving patient care and outcome in these debilitated patients.
